# Bioactive Surfaces of Polylactide and Silver Nanoparticles for the Prevention of Microbial Contamination

**DOI:** 10.3390/ma13030768

**Published:** 2020-02-07

**Authors:** Oana Gherasim, Alexandru Mihai Grumezescu, Valentina Grumezescu, Florin Iordache, Bogdan Stefan Vasile, Alina Maria Holban

**Affiliations:** 1 Department of Science and Engineering of Oxide Materials and Nanomaterials, Faculty of Applied Chemistry and Materials Science, Politehnica University of Bucharest, 011061 Bucharest, Romania; oana.fufa@gmail.com (O.G.); grumezescu@yahoo.com (A.M.G.); bogdan.vasile@upb.ro (B.S.V.); 2 Lasers Department, National Institute for Lasers, Plasma and Radiation Physics, 077125 Magurele, Romania; 3 Biochemistry Department, Faculty of Veterinary Medicine, University of Agronomic Sciences and Veterinary Medicine of Bucharest, 011464 Bucharest, Romania; floriniordache84@yahoo.com; 4 Microbiology & Immunology Department, Faculty of Biology, University of Bucharest, 077206 Bucharest, Romania; alina_m_h@yahoo.com

**Keywords:** silver nanoparticles, laser processing, bioactive coatings

## Abstract

Thanks to its peculiar interactions with biological molecules and structures, metallic silver in the form of silver nanoparticles achieved a remarkable comeback as a potential antimicrobial agent. The antimicrobial use of silver nanoparticles is of clinical importance, as several pathogenic microorganisms developed resistance against various conventional drug treatments. Hence, given the extensive efficiency of silver nanoparticles against drug-sensitive and drug-resistant pathogens, their therapeutic implications were demonstrated in multiple medical applications, such as silver-based dressings, silver-coated biomedical devices and silver-containing nanogels. Bacterial strains possess an intrinsic ability to form well-organized microbial communities, capable of developing adaptive mechanisms to environmental aggression and self-protective pathways against antibiotics. The formation of these mono- or poly-microbial colonies, called biofilms, is closely related with the occurrence of infectious processes which result in severe and chronic pathologies. Therefore, substantial efforts were oriented to the development of new protective coatings for biomedical surfaces, capable of sustaining the physiological processes within human-derived normal cells and to disrupt the microbial contamination and colonization stages. Nanostructured materials based on polylactic acid and silver nanoparticles are herein proposed as bioactive coatings able to prevent the formation of microbial biofilms on biomedical relevant surfaces.

## 1. Introduction

Conventional antimicrobial therapy represents the general choice in treating infectious diseases, despite its partial selectivity and relative specificity. The limitations or drawbacks related to this traditional drug-based therapeutic strategy mainly rely on: (i) the inappropriate prescription and inadequate administration of antimicrobial agents; (ii) the reduced therapeutic efficiency and accompanying side effects; (iii) the alarming occurrence of well-organized microbial communities and drug-resistant pathogens [1,2]. 

The development and clinical implementation of new and efficient antimicrobial agents imply intensive and incessant hardworking and extremely long-lasting and expensive protocols [3,4] The opportunistic contamination and circumstantial colonization of tissues and implanted medical devices significantly affect the patient’s quality of life. In particular, the clinical implications related to biofilm-embedded microorganisms include moderate and unspecific symptomatology, severe and chronic infections, high resistance and persistence to generally effective antimicrobials [5,6]. In the alarming context of microbial biofilm-associated infections, international healthcare professionals and researchers turned their attention towards re-evaluating and perfecting antimicrobial therapeutic alternatives.

In this context, silver-based compounds and materials proved to be promising candidates for unconventional and efficient anti-infective treatments. This particular biomedical use of silver derivatives and structures relies on their intrinsic specific and selective antimicrobial mechanisms and efficient actions against a wide range of pathogens susceptible or resistant to common drugs [7,8].

Silver nanoparticles (AgNPs) reported an impressive comeback as efficient antimicrobial agents, being already successfully applied in various biomedical, environmental and industrial technologies, and products used in everyday life. Silver-based nanosized and nanostructured materials are considered promising systems and platforms for modern applications in biomedicine and biotechnology, mainly thanks to their high antimicrobial efficiency [9,10]. Nano-silver proved efficient biocide or biostatic effects against planktonic and sessile pathogenic cells [11,12,13], promising data being reported against Gram-positive and Gram-negative bacteria [14,15], fungi and yeasts [16,17], but also viruses [18,19]. 

Although the antibacterial mechanisms of AgNPs have been extensively discussed, their exact effects on bacteria are still undefined. First of all, AgNPs can anchor to the bacterial cell wall and consequently infiltrate it. This action further causes physical changes in the bacterial membrane, such as membrane damage, which can lead to cellular content leakage and bacterial death [20]. Another antibacterial mechanism of AgNPs is related to their ability of producing high levels of reactive oxygen species (ROS) and free radical species, such as hydrogen peroxide, superoxide anion, hydroxyl radical, hypochlorous acid and singlet oxygen [21,22]. Under normal circumstances, ROS generated in cells is limited and can be eliminated by antioxidant systems. Moreover, AgNPs exert antibacterial effects through inactivation of respiratory chain dehydrogenases or alteration of other vital macromolecules and eventual excess ROS generation, which inhibit respiration and cellular growth [23,24].

In terms of biocompatibility and antimicrobial efficiency, silver nanostructures represent a suitable choice towards inducing or potentiating the antimicrobial effects of general use biomedical devices. The biological behavior of AgNPs can be tuned by microstructural and functional modifications (such as morphology and size, respectively reactivity and surface chemistry) [25,26], as to encourage beneficial interactions with normal and healthy cells and tissues. The intrinsic antimicrobial activity related to nano-silver and AgNPs-containing materials can be applied in medicine for the prevention and control of pathogenic contamination and colonization on medical devices [27,28], reduction and elimination of wound-associated infections [29,30], as well as decontamination and disinfection in water treatment [31,32]. 

Surfaces modified or coated with nano-silver structures may reduce microbial attachment and biofilm formation [24], being intensively investigated for the development of new and performance-enhanced medical devices. 

Poly(lactic acid) or polylactide (PLA) is a versatile thermoplastic biopolymer derived from natural and renewable resources, with impressive potential for clinical applications. The intrinsic features of PLA biomaterials include versatile composition and cost-effective processability, facile synthesis of blend, composite and hybrid materials and devices, adaptable mechanical and thermal behavior, adjustable permeability and solubility, excellent biocompatibility, and tunable biodegradability. 

In particular, pharmaceutical formulations based on PLA have low immunogenicity, result in non-toxic and metabolically active degradation products, and provide controlled and circumstantial release kinetics of therapeutic molecules [33,34]. Specialized and regulatory institutions approved several PLA formulations for the treatment of a few human conditions (periodontal disease, endometriosis and fibrosis, breast and prostate cancers) [35,36].

Thanks to its attractive properties and versatile biofunctionality, significant attention was oriented to the development and optimization of PLA-based biomaterials and devices for unconventional antimicrobial therapy. Promising synergetic effects (anti-infective action combined with restorative, regenerative, anti-inflammatory, immunomodulatory, or anti-tumor activity) were reported by using micro- and nanoparticles [37,38], fibers [39], films [40], scaffolds [41], and hydrogels [42] based on or containing polylactide. 

The development of anti-infective coatings able to diminish or eliminate infections related to implantable biomaterials and prosthetic devices and to combat the antimicrobial tolerance or resistance phenomena is of great interest for the biomedical field. This coating-based approach to control the adherence, contamination, and colonization stages of pathogenic strains could provide both (i) new tools to study the microbial virulence and biofilm formation mechanisms, and (ii) approaches to design film-coated surfaces for solid materials, which prevent or disrupt the formation of microbial biofilms [43,44]. 

Intravenous catheters provide vital support for patients undergoing hospitalization, including monitoring, hydration and nutrition, and drug administration. However, they are prone to pathogenic contamination and colonization, being responsible for nosocomial infections and moderate to severe healthcare complications [45,46]. Therefore, impressive efforts are oriented towards the surface modification of such medical devices, as to induce or potentiate their anti-infective activity (from antifouling to biostatic and biocide actions). 

By using the matrix assisted pulsed laser evaporation (MAPLE) technique we herein prepared PLA/AgNPs coatings. The nanostructured composite coatings proved biocompatible and exhibited antimicrobial and biofilm modulation potential, representing promising materials to be applied for surface modification of general use medical devices.

## 2. Materials and Methods

### 2.1. Materials

All chemicals used for the synthesis of nanostructured materials were purchased from Sigma-Aldrich Chemie GmbH (Taufkirchen, Germany), specifically silver nitrate (AgNO_3_), D-glucose (C_6_H_12_O_6_), sodium stearate (C_18_H_35_NaO_2_), sodium hydroxide (NaOH), sodium chloride (NaCl), poly(D,L-lactide) (PLA), and dimethyl sulfoxide (DMSO).

Double side polished (1 0 0) silicon (Si) substrates and uncoated polyvinyl chloride (PVC) central venous catheters (4 mm outer diameter) were provided by a local supplier.

Several reagents used for cellular assays were also provided by Sigma-Aldrich, namely Dulbecco’s Modified Eagle’s Medium (DMEM), Luria broth (LB), antibiotic mixture, fetal bovine serum (FBS) and phosphate buffer saline (PBS). Vybrant^®^ MTT Cell Viability Assay Kit and CMTPX fluorescent dye were supplied by ThermoFischer Scientific (Waltham, MA, USA).

EA.hy926 endothelial cells (ATCC^®^ CRL-2922), and *Staphylococcus aureus* (ATCC^®^ 25923) and *Escherichia coli* (ATCC^®^ 25922) strains were purchased from American Type Culture Collection.

### 2.2. Synthesis Methods

#### 2.2.1. Synthesis of AgNPs

The synthesis of AgNPs was performed using a facile reduction protocol, which required the use of (i) an inorganic solution (the metallic precursor solution was obtained by dissolving AgNO_3_ in ultrapure water) and (ii) an organic solution (the reducing/stabilizing solution was obtained by dissolving D-glucose and sodium stearate in alkaline ultrapure water, respectively). The Ag-containing solution was dropwise added to the organic solution, under continuous stirring. A stable suspension, which gradually turned dark grey, resulted after completion of the synthesis process. NaCl was added to the final suspension for destabilization and subsequent decantation of Ag-based precipitate. The Ag-containing slurry was collected by filtration, triply washed and dried at room temperature, resulting in a silver-based powdery sample.

#### 2.2.2. Synthesis of PLA/AgNPs Coatings

Prior to MAPLE experiments, the substrates intended for surface modification were subjected to a thorough ultrasonic cleaning treatment. The PLA/AgNPs materials were deposited on (1 0 0) silicon substrates with 1 cm^2^ area for physicochemical investigations. For complementary biological and microbiological studies, cylindrical catheter sections with 1 cm length were used, the MAPLE experiments aiming to modify their external surface. 

The PLA/AgNPs coatings were obtained using the MAPLE method, by using a KrF* excimer laser source (λ = 248 nm, τ_FWHM_ = 25 ns), model COMPexPro 205 Lambda Physics from Coherent (Göttingen, Germany). In this respect, solid targets were obtained by freezing the suspensions of PLA and AgNPs in DMSO (1.5% concentration), at liquid nitrogen (LN) temperature (~77 K). The composite targets were irradiated at different laser fluences (300, 400 and 500 mJ/cm^2^), by applying 50,000 laser pulses. Several experimental parameters were maintained constant, regardless the laser fluence, such as room temperature and 0.1 Pa pressure within the deposition chamber, 5 cm target—substrate distance, 0.4 Hz target rotation frequency, 15 Hz laser repetition frequency. 

### 2.3. Physicochemical Characterization

#### 2.3.1. X-ray Diffraction (XRD) 

In terms of purity and crystallinity, the Ag-based powdery sample was investigated by XRD, using in this respect an XRD-6000 Shimadzu diffractometer (Duisburg, Germany). The scans were collected at room temperature, using Cu_Kα_ radiation (λ = 1.056 Å) in the 10–80° angle range. 

#### 2.3.2. Transmission Electron Microscopy (TEM)

The TEM images were collected with a Tecnai^TM^ G2 F30 S-TWIN high resolution transmission electron microscope from FEI Company (Hillsboro, OR, USA). The instrument operated in the transmission mode at 300 kV, with point and line resolutions of 2 and 1 Å. 

By using the selected area electron diffraction (SAED) accessory of the microscope, complementary crystallographic information was obtained.

#### 2.3.3. Infrared Microscopy (IRM)

Compositional data on the MAPLE processed materials were obtained by complementary infrared mappings and spectra. Investigations were performed with a Nicolet iN10 MX Fourier transform (FT)-IR microscope from ThermoFischer Scientific. The scans were performed in the reflection mode with 4 cm^−1^ resolution, in the range 4000–600 cm^−1^. For each sample, 32 individual scans were recorded, co-added, and converted to absorbance using the OmincPicta software (Version 8.0, ThermoFischer Scientific).

#### 2.3.4. Scanning Electron Microscopy (SEM)

For microstructural analysis, the MAPLE samples were subjected to SEM investigations, following their capping with a thin Au conductive layer. In this respect, the secondary electron beam (30 keV) of an electronic microscope from FEI was used. 

Additional information on the composition was obtained using the energy dispersive X-ray spectroscopy (EDS) accessory of the microscope. 

### 2.4. Biological Evaluation 

#### 2.4.1. MTT Cell Proliferation Assay 

The biological behavior of central venous catheter sections modified with PLA/AgNPs coatings was assessed on EA.hy926 cells. The endothelial cells were maintained in DMEM supplemented with FBS and antibiotic mixture in standard conditions (37 °C, 5% CO_2_ and >90% humidity). Prior to cellular tests, the MAPLE processed samples were sterilized by UV exposure.

The cytotoxicity of PLA/AgNPs-coated catheter sections was assessed using the MTT assay (Vybrant^®^ MTT Cell Viability Assay Kit). Based on this quantitative colorimetric method, cell viability, cytotoxicity, genotoxicity, and evaluation of anticancer drugs are allowed [47,48,49]. The viable cells reduce the yellow tetrazolium salt MTT (3-(4,5-dimetiltiazoliu)-2,5-diphenyltetrazolium bromide) to a dark blue formazan. The reduction is made by mitochondrial enzymes (especially succinate dehydrogenase) and is an indicator for cellular / mitochondrial integrity. The as-resulted water-insoluble formazan can be solubilized with isopropanol, dimethyl sulfoxide, or other organic solvents. The optical density (OD) of the solubilized formazan is spectrophotometrically evaluated, obtaining a function absorbance-concentration dye-umber of metabolically active cells in the culture. 

Cells were cultured in 96-well plates, at a seeding density of 3000 cells/well. After their incubation in different experimental conditions (24 and 48 h), a volume of 10 μL 12 mM MTT solution was added and the cultures were additionally incubated at 37 °C for 4 h. Subsequently, 100 µL of SDS-HCl solution was added, incubated for 1 h and vigorously pipetted for the solubilization of formazan crystals. The absorbance was spectrophotometrically read at 570 nm with an Infinite M200 instrument from TECAN (Männedorf, Switzerland). 

#### 2.4.2. Fluorescence Microscopy

The biocompatibility of nanostructured surfaces was also evaluated by fluorescence microscopy, using the CMTPX dye, a cell tracker for long-term tracing of living cells. The CMTPX compound was added in cell culture and the viability and morphology of EA.hy926 endothelial cells was evaluated after 5 days. The CMTPX red fluorophore was added in the culture medium at a final concentration of 5 μM, followed by 30 min of incubation, in order to allow penetration of the dye into the cells. Next, the EA.hy926 endothelial cells were washed with PBS and visualized by fluorescence microscopy. The photomicrographs were taken with Olympus CKX 41 digital camera driven by CellSense Entry software (Olympus, Tokyo, Japan).

#### 2.4.3. Cell Cycle Analysis 

The cell cycle analysis was performed by flow cytometry. The EA.hy926 endothelial cells were incubated for 48 h with the PLA/AgNPs-coated catheter sections, then harvested and washed with PBS. The cell pellet was fixed in cold 70% ethanol for 30 min at 4 °C, followed by a double washing treatment with PBS, interspersed by two centrifugations at 400× *g*. The cell pellet was treated with 50 μL of ribonuclease (100 μg/mL concentration) and 200 μL of propidium iodide (50 μg/mL concentration). The samples were analyzed with the Gallios Flow Cytometer from Beckman Coulter (Indianapolis, IN, USA) and the results were obtained using Gallios software (version 1.0).

#### 2.4.4. Data Analysis 

All experiments described in this study were performed using three biological replicates. The technical replicate depended on the experiment type and varied from 2–4 replicates. Data were reported as mean ± SD, where “n” indicates the number of samples. The statistical significance was assessed using two-tailed Student’s *t* test. For all statistical tests we set a critical level *p* = 0.05.

### 2.5. Microbiological Evaluation

To assess the ability of PLA/AgNPs coatings to interfere with the formation of bacterial biofilms, glycerol stocks of *S. aureus* and *E. coli* strains were used. Prior to antimicrobial tests, all samples (uncoated and PLA/AgNPs-coated catheter sections) were sterilized by UV exposure for 20 min. 

Each piece of sterile material was individually placed in one well of 6-well sterile plates (Nunc), followed by the addition of 2 mL of LB medium. 50 μL of bacterial suspensions of 0.5 McFarland (1.5 × 10^8^ CFU (colony-forming units)/mL) standard densities were inoculated in each well. The as-prepared plates were incubated at 37 °C for 24 h. The samples were subsequently washed with sterile phosphate buffered saline (PBS), in order to remove unattached cells. In the next step, the samples were transferred in sterile tubes containing 1 mL of fresh PBS and subjected to vigorous vortexing for 30 s to detach the microbial cells from biofilms. The obtained cell suspensions were diluted and a volume of 10 µL from each dilution was seeded in triplicate onto LB agar plates. After 24 h of incubation in standard conditions, the colony-forming units (CFU/mL) values were determined by viable cells count [50,51]. Each experiment was performed in triplicate and repeated on three separate occasions.

Data were analyzed using GraphPadIn Stat and Prism softwares (Version 6.01, GraphPad Software Inc., San Diego, CA, USA), by applying the One-way Analysis of Variance (ANOVA) test. *p* values lower than 0.05 were considered significant.

## 3. Results and Discussions

### 3.1. Physicochemical Investigation of AgNPs

**Figure 1:**
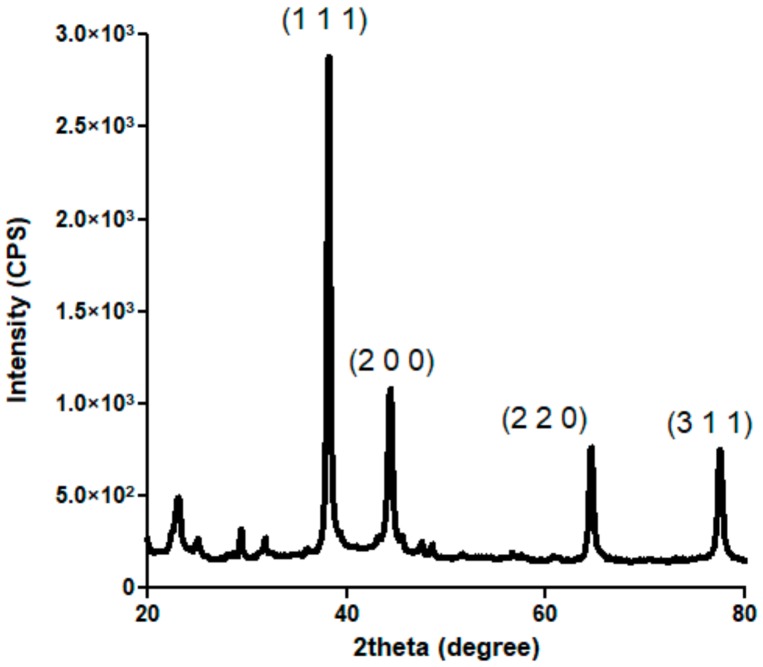
XRD diffractogram of AgNPs.

Figure 1 presents the X-ray diffractogram recorded for the obtained silver-based powdery sample. Intense diffraction maxima are identified at 2θ values of ~38°, ~45°, ~65°, and ~78°. According to the available specialized data (PDF no. 00-004-0783), these peaks correspond to (1 1 1), (2 0 0), (2 2 0) and (3 1 1) diffraction planes of silver crystals with face-centered cubic (fcc) lattice [52,53]. By indicating the high purity and crystalline nature of silver-based sample and by showing its characteristic fcc crystallographic lattice, the XRD pattern confirms that metallic silver represents the sole crystalline phase of the obtained powder.

**Figure 2:**
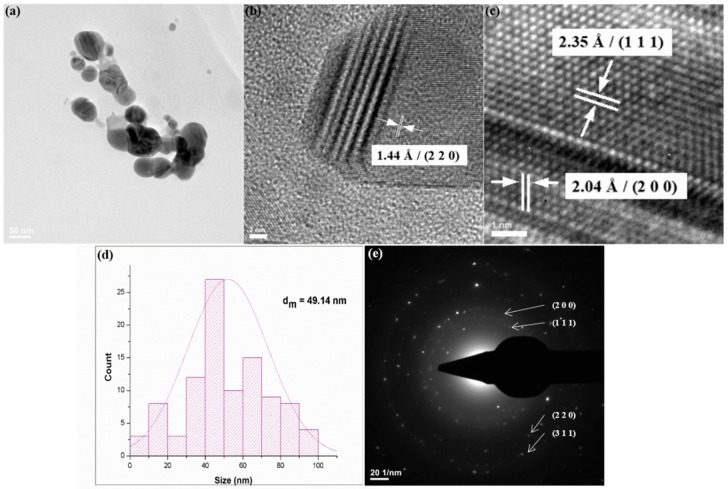
TEM micrograph (**a**), HR-TEM images (**b**,**c**), particle size histogram (**d**) and SAED pattern (**e**) of AgNPs.

The TEM micrograph from Figure 2a confirms the nanosized dimension of obtained silver particles, with mean particle size of ~49 nm (Figure 2d). Also, a general tendency of AgNPs to form aggregates is shown, as well as their quasi-spherical morphology. The preferential shape can also be noticed in the HR-TEM images. The previously identified crystalline planes and interplanar distances are shown in the HR-TEM micrographs (Figure 2b,c). The SAED spectrum from Figure 2e confirms previous XRD data on the crystalline nature and crystal structure of AgNPs.

### 3.2. Physicochemical Investigation of PLA/AgNPs Coatings 

**Figure 3:**
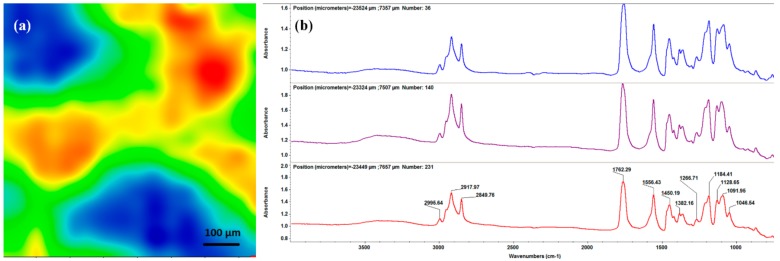
IR map (**a**) and IR spectrum (**b**) of PLA/AgNPs dropcast.

**Figure 4:**
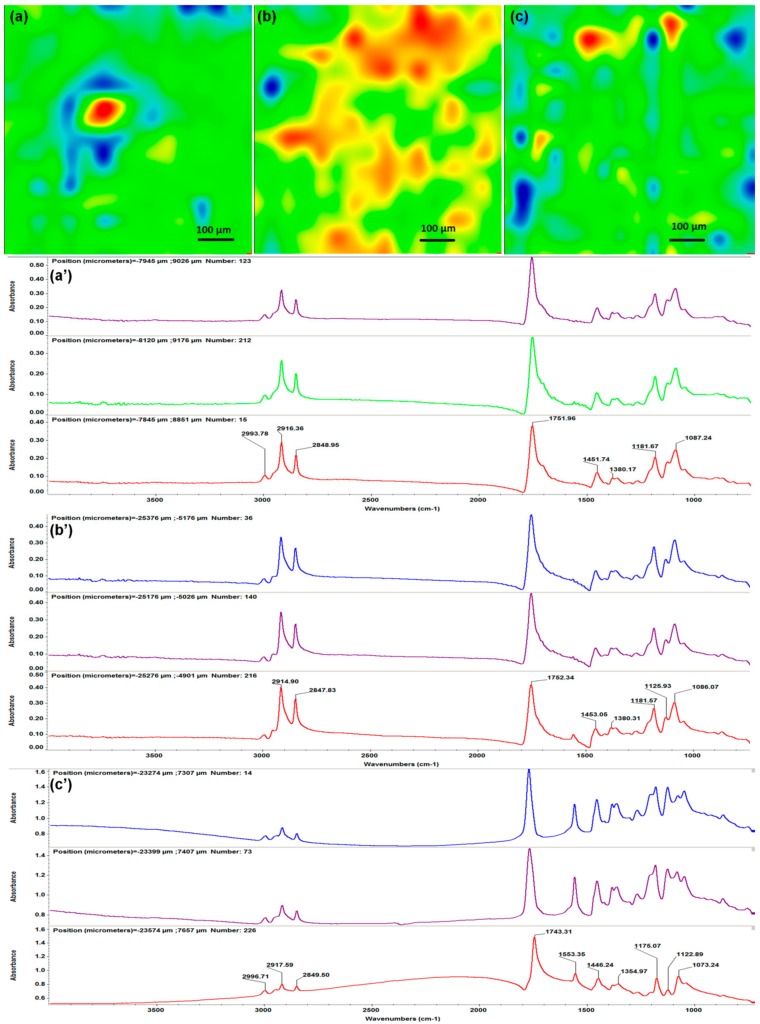
IR maps (**a**–**c**) and IR spectra (**a’**–**c’**) of PLA/AgNPs coatings obtained at laser fluences of 300 mJ/cm^2^ (**a**,**a’**), 400 mJ/cm^2^ (**b**,**b’**) and 500 mJ/cm^2^ (**c**,**c**’).

Infrared analysis was performed in order to evaluate compositional effects of the laser processing on the PLA/AgNPs material. In this respect, the IR data of dropcast sample (corresponding to pristine material, Figure 3) was compared to the IR data of MAPLE samples (Figure 4). The infrared maps (Figure 3a and Figure 4a–c) were obtained by monitoring the distribution of C–H absorption band (common for both polymer and fatty acid salt). IR colors depend on the absorbance intensity: the blue to green areas indicate an insignificant to minimum intensity, while the red to orange areas indicate a maximum to reduced intensity, respectively. The corresponding infrared spectra (Figure 3b and Figure 4a’–c’) were collected from different points of the samples. 

To evaluate the optimal experimental conditions for MAPLE processing of PLA/AgNPs materials, the infrared data from the dropcast sample (Figure 3) were used as reference. As mentioned, the mapping was performed by monitoring the C–H bond, which presence is noticed in the IR spectrum (Figure 3b) in the 2800–3000 cm^−1^ wavenumber range. Strong stretching vibrations of C–H are noticed at ~2995 cm^−1^ (originating from –CH_3_ terminal groups of both organic compounds), ~2918 and ~2850 cm^−1^ (asymmetric and symmetric vibrations of –CH_2_ from the long chain of sodium stearate, respectively) [54,55]. Common IR maxima are also identified at ~1762 cm^−1^ (strong stretching vibrations of C=O), ~1450 cm^−1^ (overlapping of asymmetrical bending of terminal C–H and symmetrical stretching of –COO^-^ function from stearate) and ~1091 cm^−1^ (strong stretching vibrations of C–O) [56,57,58]. Bending and asymmetric vibrations of –CH_3_ and C–O–C bonds from PLA are present at ~1382 and ~1184 cm^−1^, respectively [59,60]. The infrared bands at ~1556 and ~1046 cm^−1^ are characteristic for sodium stearate, corresponding to asymmetric stretching vibrations of the carboxylate group, respectively to rocking vibrations of the methylene function [61,62].

The IR data corresponding to PLA/AgNPs materials processed with different laser fluences are included in Figure 4. The infrared maps of samples obtained with laser fluences of 300 and 500 mJ/cm^2^ (Figure 4a,c) evidence the improper transfer of PLA/AgNPs materials. In terms of composition, predominant cool areas indicate non-uniform coatings. Complementary information is obtained by considering the IR spectra (Figure 4a’,c’ respectively). In comparison with previous results (Figure 3b), the lowest laser fluence (300 mJ/cm^2^) did not affect the chemical structure of PLA/AgNPs material (Figure 4a’). Slight modifications on the absorption of IR maxima are noticed, indicating that an insufficient amount of material was transferred onto the substrate at this laser fluence. Contrariwise, IR spectra of the 500 mJ/cm^2^ MAPLE experiment (Figure 4c’) evidence significant modifications of absorption bands. Severe intensity decreases and position shifts can be observed, indicating that the laser beam damaged the organic material. 

The IR map of PLA/AgNPs material processed with the middle laser fluence (Figure 4b) shows uniform distribution of chemical functions and complete coverage of the substrate. Additionally, the corresponding spectra (Figure 4b’) mark the presence of all above-mentioned absorption maxima, with no signs of bonds damage. 

Based on the infrared data, we selected the 400 mJ/cm^2^ laser fluence as the optimal choice for MAPLE processing of PLA/AgNPs materials, in terms of compositional integrity and efficient transfer. Therefore, SEM analysis and cellular evaluation were performed on the PLA/AgNPs coatings obtained at 400 mJ/cm^2^ laser fluence.

**Figure 5:**
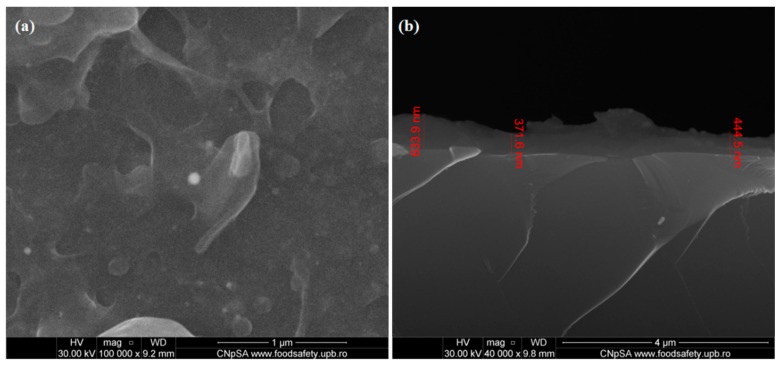
Plain view (**a**) and cross section (**b**) SEM images of PLA/AgNPs coatings.

Microstructural data on the PLA/AgNPs coatings obtained with 400 mJ/cm^2^ laser fluence are presented in the SEM micrographs from Figure 5. The plain view image (Figure 5a) shows the formation of a continuous coating onto the substrate. It can be noticed that AgNPs are embedded within the polymer matrix and the composite coating exhibits irregular surface morphology, as further confirms the cross section micrograph (Figure 5b). The thickness of PLA/AgNPs coatings is of ~500 nm. 

The EDS spectrum (data not shown) evidenced the predominant presence of metallic Ag (specific triple absorption lines positioned at ~3 keV), followed by carbon and silicon (originating from the substrate). This indicates the successful MAPLE synthesis of high purity PLA/AgNPs coatings. 

### 3.3. Biological Evaluation of PLA/AgNPs Coatings

The biological response of central venous catheters modified with PLA/AgNPs nanostructured coatings obtained with 400 mJ/cm^2^ laser fluence was evaluated in the presence of EA.hy926 endothelial cells by MTT assay, fluorescence microscopy and flow cytometry. 

**Figure 6:**
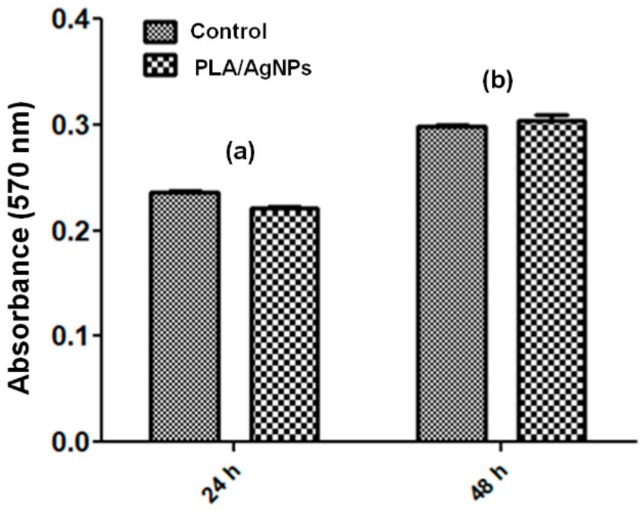
Viability results of human endothelial cells cultured for 24 h (**a**) and 48 h (**b**) on composite PLA/AgNPs coatings using the MTT assay (n = 3, * *p* < 0.05).

Our results show that the composite coatings did not have cytotoxic effects on normal human-derived eukaryotic cells. The MTT assay demonstrates that human endothelial cells have a normal metabolism and growth in the presence of PLA/AgNPs coatings, as the measured values of absorbance at 570 nm are comparable with those of control cells (Figure 6). Analyzing the results, it is observed that there are no notable differences after at 24 h and 48 h between control samples and PLA/AgNPs-coated samples. After both time intervals, the decrease of cellular activity recorded for samples coated with nanostructured composite was assigned with *p* > 0.05 (in comparison with uncoated control catheter sections). Therefore, PLA/AgNPs materials proved to represent non-cytotoxic with respect to EA.hy926 cells, as indicated by the statistically non-significant modifications of cellular viability.

**Figure 7:**
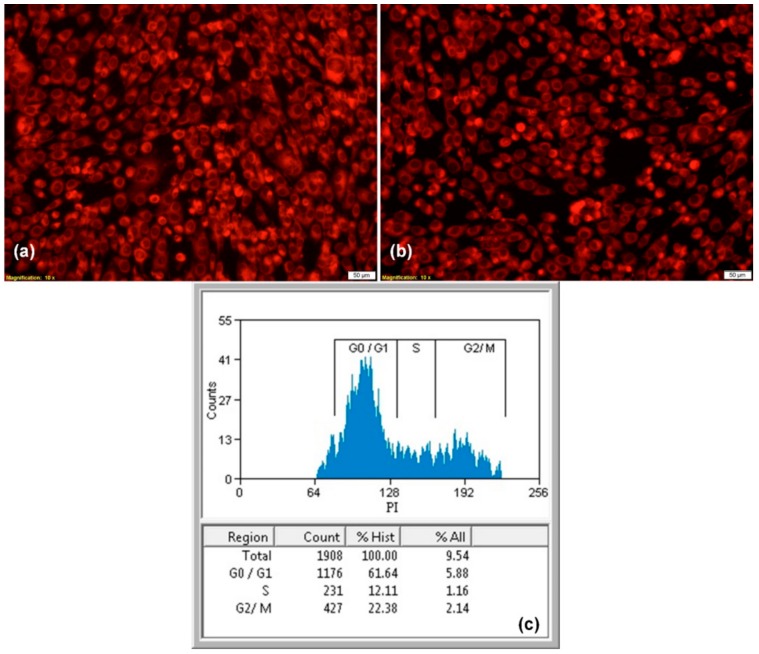
Fluorescence micrographs of human endothelial cells cultured after 5 days on control (**a**) and composite PLA/AgNPs coatings (**b**), 50× magnification; the corresponding cell cycle after 48 h of incubation (**c**) (n = 3, * *p* < 0.05).

After 5 days of incubation in the presence of nanostructured coatings, human endothelial cells presented a normal morphology with endothelial-like phenotype. The fluorescent images show that endothelial cells cultured on PLA/AgNPs-coated samples present similar features with control cells, being viable, without dead cells or cell fragments, spreading filopodia and establishing contacts with neighboring cells, suggesting that endothelial cells have an active phenotype (Figure 7a,b).

To study the effects of composite coatings in cell cycle activity of human endothelial cells, flow cytometry was employed. Cell cycle anomalies are observed after different types of cell damages, leading to arrest of the cell cycle progression in a certain phase, G0/G1, S or G2/M. In our experiments, we examined whether the PLA/AgNPs coatings modify cell cycle or induce cell cycle arrest in endothelial cells. The results show that the nanostructured surfaces did not modified the cell cycle, with a characteristic profile for adult cells, with 61.64% in G0/G1 phase, 12.11% in the S phase and 22.38% in the G2/M phase (Figure 7c). 

In our previous studies, we demonstrated that the intraperitoneal inoculation of 200 μL of AgNP suspension does not determine histological modification and inflammatory alteration in brain, myocardium, pancreas, liver, lung, and kidney tissues after 7 days and 14 days of experimental treatment [63]. In another study, we showed that the synthesis method of nanoparticles is important in relation to cellular biocompatibility. AgNPs synthesized through pyrosol method, at 650 °C and 700 °C, proved extremely toxic for the mesenchymal stem cells, inducing significant changes in cellular morphology and viability. AgNPs synthesized through sol–gel techniques proved good biocompatible, since they did not influence the viability of eukaryotic cells and, moreover, the cloning assay proved a high efficiency [64].

In conclusion, the overall results of biological assays suggest that catheter sections modified with PLA/AgNPs composite coatings are biocompatible with human endothelial cells and can be used for biomedical applications.

### 3.4. Microbiological Evaluation of PLA/AgNPs Coatings

The ability of PLA/AgNPs coatings to inhibit the dynamic formation of monospecific biofilms was assessed against *S. aureus* and *E. coli* opportunistic pathogens. 

It has been demonstrated that AgNPs may induce a dose-, size- and time-dependent cytotoxicity, particularly if the size is ≤10 nm. In the same time, their antimicrobial efficiency is widely recognized. Therefore, AgNPs are considered a double-edged sword that can diminish or eliminate pathogenic microorganisms, but induce cytotoxicity in mammalian cells [65,66].

**Figure 8:**
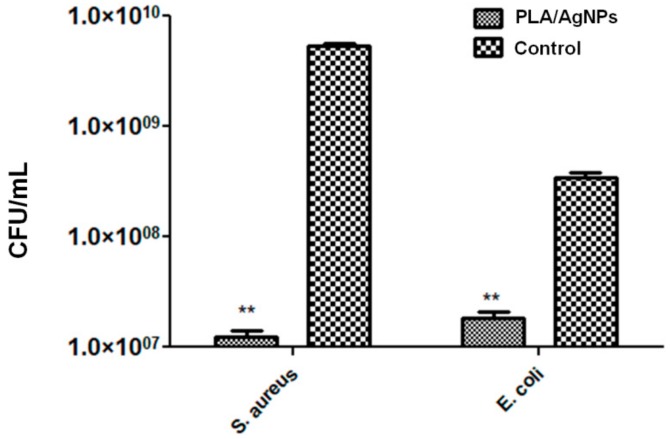
Microbial biofilm development of *S. aureus* (left) and *E. coli* (right) strains after 24 h of incubation with composite PLA/AgNPs coatings.

The microbiological results are included in Figure 8 and demonstrate that PLA/AgNPs coatings significantly interfere with the development of bacterial biofilms. The process was especially inhibited in the case of Gram-positive strain (Figure 8 (left)). In comparison with unmodified specimens, the bacterial population is reduced with more than 2.5 orders of magnitude (logs). Even if noticeably reduced, the efficiency of PLA/AgNPs coatings against the development of Gram-negative colonies is reduced with ~1.2 order of magnitude (Figure 8 (right)). 

The microbiological results show the efficiency of PLA/AgNPs coatings to inhibit the first stages of bacterial biofilm formation (i.e., attachment, microcolony formation), but also the maturation phase. The proposed nanostructured materials could represent a feasible strategy towards modifying the surface of short-term to mid-term implantable devices.

## 4. Conclusions

Nanostructured and uniform coatings based on PLA and quasi-spherical crystalline AgNPs were obtained by MAPLE. Commercial catheter sections modified with PLA/AgNPs proved biocompatible behavior, being able to support the proliferation and normal growth of human endothelial cells *in vitro*. The composite coatings significantly inhibited the formation of monospecific biofilms of two relevant opportunistic pathogens: *S. aureus* and *E. coli*, with an increased efficiency against the Gram-positive strain. Composite and nanostructured coatings represent a feasible strategy to modify the surface of biomedical devices by means of biocompatible materials, which additionally can induce or potentiate anti-infective effects.
